# Supplementary material for: Sources of dehydration fluids underneath the Kamchatka arc
Source: Nat Commun. 2022 Aug 2;13:4467. doi: 10.1038/s41467-022-32211-5 (PMC9345910; doi:10.1038/s41467-022-32211-5)
Supplement: Supplementary file 1 — Supplementary Information [file 41467_2022_32211_MOESM1_ESM.pdf]

Supplementary information

Sources of dehydration fluids underneath the Kamchatka arc

Yunchao Shu[1,2,3,*], Sune G. Nielsen[1,4,*], Veronique Le Roux[4], Gerhard Wörner[5], Jerzy Blusztajn[1,4], Maureen Auro[1]

[1]NIRVANA (Non-traditional Isotope Research for Various Advanced Novel Applications) Laboratories, Woods Hole Oceanographic Institution, Woods Hole, MA 02543, USA

[2]The Pheasant Memorial Laboratory for Geochemistry and Cosmochemistry, Institute for Planetary Materials, Okayama University, Misasa, Tottori 682-0193, Japan

[3]CAS Key Laboratory of Crust-Mantle Materials and Environments, School of Earth and Space Sciences, University of Science and Technology of China, Hefei 230026, China

[4]Department of Geology and Geophysics, Woods Hole Oceanographic Institution, Woods Hole, MA 02543, USA

[5]Geowissenschaftliches Zentrum der Universität Göttingen, Göttingen 37073, Germany

Supplementary Note 1: Degassing

Before or during eruption the thallium budgets of Kamchatka arc lavas may be affected by magma degassing because thallium is a highly volatile metal[1,2], which can result in kinetic isotope fractionation with heavy thallium isotope compositions developing with increasing degree of degassing[3]. Significant degassing of Tl should be manifested as significant depletions of Tl relative to refractory elements with similar compatibility during mantle melting such as Ce[3-5]. Given that Ce/Tl ratios in both sediments (Ce/Tl ~ 150) and HESC samples (Ce/Tl = ~550) are much lower than that of the upper mantle (Ce/Tl ~ 1100[5]), it could be inferred that unusually high Ce/Tl ratios, especially those exceeding the mantle value, coupled with heavy Tl isotope compositions might result from significant magma degassing. A recent study conducted the first experimental investigation of the effects on lavas of thallium degassing[3]. The simulated degassing curves fit very well with the variations of Ce/Tl ratios and Tl isotope compositions for Bakening, Gamchen and Komarov volcanos[3]. To assess the thallium isotopic modification induced by degassing, we use the kinetic degassing model deduced from degassing experiments[3], for each volcano from Kamchatka arc that we studied here. The theoretical degassing trends are shown in Supplementary Figure 2 and reveal that in addition to Bakening, Gamchen and Komarov volcanos (Supplementary Fig. 2a, b and c), samples from Kluchevskoy, Esso and Shmidt also exhibit patterns that are consistent with kinetic isotope fractionation during degassing (Supplementary Fig. 2g, i and e). Kizimen, Nikolka, Tolbachik, Ichinsky and Achtang volcanos do not display any clear relationships between Ce/Tl ratios and thallium isotope compositions (Supplementary Fig. 2d, h, f, j and k), although one sample from Kizimen exhibits unusually high Ce/Tl and a positive Tl isotope value, that is likely associated with degassing as well.

In order to remove any doubt regarding the sub-arc mantle origin of the measured Tl isotope compositions we exclude degassed samples from Bakening, Gamchen, Komarov, Kluchevskoy, Esso, Shmidt and Kizimen volcanos and use pre-degassing samples of each volcano for our discussion of slab-mantle interaction in the Kamchatka arc subduction zone. We note here that Ichinsky lavas generally exhibit higher Ce/Tl ratios than other Sredinny Range lavas (Supplementary Fig. 2j), which may imply significant thallium degassing. However, as discussed in the main text, some of the Ichinsky lavas are characterized by high Ce/Tl and likely contain an OIB component in the source. Samples with elevated Ce/Tl also typically display elevated Dy/Yb, which further reflect the contribution of garnet in their source (Supplementary Fig. 3). Since samples with elevated Dy/Yb and/or elevated Ce/Tl do not reflect processes of slab material transfer in the Kamchatka subduction zone[6], we only use Ichinsky lavas with Dy/Yb ratios similar to other Kamchatka arc lavas in our assessment of

degassing (Dy/Yb < 2). These samples reveal no relationship between Ce/Tl and Tl isotopes (Supplementary Fig. 2j).

An additional observation of interest is that most of the very positive ε^{205}Tl-values are associated with lavas that have the lowest SiO_2 contents (Supplementary Fig. 4a), suggesting that degassing is more prominent for lavas with lower viscosities. This observation is generally consistent with higher degassing efficiency that has been shown to occur in lavas with low viscosity[7]. However, Tl-degassing also depends on other parameters such as magma redox state and concentrations of ligand-forming elements such as Cl and S in the magma[3].

Supplementary Note 2: Post eruption alteration

Subaerial basalt alteration has been shown to create systematically low alkali element concentrations due to rainwater leaching[8,9]. Given that thallium often behaves similarly to alkali metals K, Rb and Cs due to their similar ionic radii and charge[10-12], significant subaerial aqueous alteration can result in high Ce/Tl (> 2000) and Th/Rb (> 0.2) ratios because of the immobile nature of Ce and Th during post-magmatic alteration processes[4,5,13]. All samples studied here are relatively young (Quaternary) arc lavas[6] and samples were collected specifically to avoid any signs of alteration, which suggests that subaerial alteration is likely not pervasive and strong. All Kamchatka arc lavas that have been filtered for degassing and deep OIB components exhibit Th/Rb and Ce/Tl ratios similar to unaltered lavas from other island arcs (Supplementary Fig. 5). We, therefore, consider it unlikely that Kamchatka arc lavas were affected by subaerial alteration.

Supplementary Note 3: Assimilation and fractional crystallization (AFC)

Lavas from volcanos that were not affected by magma degassing and subaerial alteration are all basalts or basaltic andesites and display a narrow range in SiO_2 contents from 49.7 to 56.0 wt%, except for two lavas exhibiting relatively high SiO_2 contents of 59.1 and 63.6 wt% (Supplementary Fig. 4b). It is hence unlikely that the lavas are pervasively affected by fractional crystallization and/or crustal contamination. The samples with low MgO contents have stronger potential to be affected by AFC processes, although their Sr isotope compositions are indistinguishable from the range observed in more primitive lavas (Supplementary Fig. 6). Thallium is highly incompatible in early crystallizing minerals, which renders Tl isotope effects from early magmatic evolution negligible[14]. However, absolute trace element concentrations (including Tl) will likely have been modified by

fractional crystallization and, therefore, we just use ratios of trace elements with same degree of incompatibility from these samples in our assessment of slab material transport processes.

Supplementary Tables

Supplementary Table 1 Major and trace element (including thallium) compositions for the Kamchatka arc lavas[a]

Sample	SiO_2	TiO_2	Al_2O_3	Fe_2O_3	FeO	MnO	MgO	CaO	Na_2O	K_2O	P_2O_5	Sc	V	Cr	Co	Ni	Rb	Sr	Y	Zr	Nb	Cs	Ba	La	Ce	Pr	Nd	Sm	Eu	Gd	Tb	Dy	Ho	Er	Tm	Yb	Lu	Hf	Ta	Tl	Pb	Th	U
**Gamchen**																																											
GAM-96-14	55.8	0.86	18.5	3.38	5.39	0.18	3.62	7.96	3.26	0.33	0.13	25	248	4.0	22	4.0	3.6	263	20	62	0.62	0.10	117	2.82	9.41	1.51	8.01	2.58	0.893	2.84	0.494	3.23	0.726	2.18	0.323	2.15	0.34	1.65	0.061	0.0096	0.980	0.21	0.14
GAM-96-16	54.9	0.80	18.1	2.59	5.79	0.17	4.23	8.44	2.86	0.85	0.11	32	238	52	25	9.0	21	257	21	81	2.0		270																	0.052			
GAM-96-28	49.8	0.83	17.9	2.59	7.96	0.20	6.24	10.9	2.37	0.66	0.13	41	313	79	40	30	11	331	17	54	0.85	0.41	327	4.38	13.1	1.95	9.47	2.72	0.878	2.69	0.441	2.79	0.609	1.79	0.257	1.68	0.26	1.45	0.075	0.026	1.68	0.58	0.32
GAM-96-07	51.6	0.85	18.6	3.43	5.73	0.18	5.35	9.12	2.72	0.49	0.12	28	230	38	32	20	9.0	278	18	61	1.0	0.28	154	3.91	11.5	1.67	8.20	2.50	0.840	2.64	0.460	2.95	0.660	1.97	0.280	1.89	0.29	1.60	0.080	0.038	2.18	0.57	0.26
GAM-96-12	54.7	0.80	19.8	2.30	4.67	0.14	2.98	8.44	3.18	0.68	0.12	21	173	10	17		14	293	25	80			214																	0.052			
GAM-96-22	52.8	0.80	17.2	2.49	6.88	0.19	5.95	9.54	2.78	0.54	0.12	34	269	87	35	46	8.0	257	19	73	1.1	0.38	199	4.20	12.7	1.78	8.49	2.56	0.850	2.69	0.470	3.06	0.690	2.08	0.310	2.08	0.32	1.99	0.070	0.048	1.78	0.60	0.28
GAM-96-26	50.6	1.0	18.5	2.87	8.48	0.20	4.94	9.93	2.60	0.61	0.11	35	359	26	38	13	9.0	268	19	61	0.90	0.53	205	3.61	10.9	1.54	7.80	2.44	0.830	2.67	0.470	3.05	0.670	2.04	0.300	1.95	0.30	1.53	0.080	0.046	1.83	0.48	0.23
**Kizimen**																																											
KIZ-96-01	63.6	0.58	16.2	2.41	3.03	0.13	2.44	5.34	3.69	1.7	0.16	15	114	17	14		38	319	16	121	4.2	1.5	676	10.2	22.4	3.32	13.5	2.89	0.953	2.58	0.359	2.28	0.550	1.46	0.197	1.39	0.24	1.91	0.21	0.27	5.30	3.2	1.5
KIZ-96-19	50.3	1.2	16.5	2.97	8.01	0.21	5.22	9.59	2.70	0.71	0.21	35	316	42	37	25	9.3	276	32	104	4.1	0.31	164	6.49	19.1	2.73	13.1	3.86	1.14	4.04	0.695	4.37	0.941	2.89	0.419	2.77	0.42	2.80	0.21	0.049	2.03	0.59	0.38
KIZ-96-24	54.7	1.0	17.4	3.07	5.49	0.17	4.16	8.18	3.18	1.1	0.17	22	246	24	27	2.0	21	328	20	99	3.1	0.79	419	6.52	16.6	2.83	12.7	3.25	1.08	3.12	0.465	3.08	0.763	1.97	0.268	1.72	0.30	2.10	0.14	0.088	2.73	1.6	0.77
KIZ-96-01/1	49.7	1.2	18.8	5.32	5.56	0.19	5.20	9.25	2.74	0.76	0.17	26	300	15	30	2.0	14	370	21	86	2.9	0.52	310	5.85	15.2	2.34	12.0	3.36	1.14	3.28	0.540	3.33	0.740	2.18	0.300	2.00	0.29	1.99	0.17	0.11	1.95	1.0	0.49
KIZ-96-24/1	51.6	1.3	17.9	4.29	5.78	0.19	4.42	9.13	3.03	0.87	0.19	33	324	21	27		16	335	23	90	3.2	0.59	323	7.02	18.1	2.59	13.1	3.91	1.27	3.53	0.630	3.88	0.750	2.23	0.340	2.38	0.32	2.17	0.19	0.095	2.63	0.91	0.61
TAM-96-01	51.4	0.83	15.8	9.37	0.430	0.19	8.43	9.23	2.72	0.73	0.23	31	221	481	36	166	15	380	16	86	2.4	0.50	358	7.62	19.0	2.69	13.1	3.72	1.11	3.29	0.520	3.38	0.670	2.06	0.320	2.05	0.31	2.27	0.19	0.018	2.15	0.91	0.45
replicate[b]																																								0.016			
**Komarov**																																											
KOM-96-02/2	53.7	0.75	16.0	1.86	6.48	0.17	7.33	9.36	2.55	0.80	0.12	35	246	309	33	89	15	219	27	86	2.0	0.96	234	3.83	9.79	1.69	10.9	2.53	0.719	3.01	0.424	2.79	0.625	2.00	0.309	1.80	0.30	2.11	0.074	0.079	2.08	0.84	0.42
KOM-96-06	51.7	0.83	16.3	2.03	7.47	0.18	8.20	9.68	2.41	0.68	0.11	37	267	368	41	119	13	240	18	74	1.1	0.59	226	3.94	9.91	1.63	8.72	2.47	0.738	2.61	0.409	2.78	0.613	1.79	0.282	1.70	0.28	1.75	0.059	0.052	1.91	0.82	0.33
KOM-96-01	52.6	0.84	17.2	2.20	7.01	0.18	6.39	9.66	2.58	0.79	0.13	33	274	137	36	35	18	231	24	84	1.5	0.78	261	4.36	11.8	1.87	10.4	2.89	0.840	3.14	0.470	3.23	0.690	2.13	0.330	2.00	0.32	2.19	0.090	0.059	2.42	0.82	0.36
KOM-96-14	53.5	0.81	17.8	3.19	6.24	0.18	5.36	9.33	2.83	0.56	0.12	30	269	11	36	24	9.0	247	21	71	1.7	0.55	174	4.54	10.4	1.40	8.98	2.78	0.830	2.88	0.490	2.98	0.630	2.12	0.320	2.02	0.30	1.93	0.080	0.037	1.75	0.52	0.27
**Shmidt**																																											
SHM-96-01	50.6	0.89	16.2	3.75	5.94	0.18	8.07	10.4	2.34	0.57	0.12	36	257	335	43	103	15	215	21	74	1.6	0.67	189	3.68	9.72	1.57	8.65	2.51	0.780	2.67	0.470	2.85	0.670	2.18	0.311	1.90	0.27	1.91	0.098	0.012	1.63	0.57	0.29
SHM-96-03	59.1	0.70	18.4	3.78	3.49	0.21	2.21	6.99	3.60	0.46	0.056	18	34.0		7.0		7.2	408	17	62	1.1	0.37	168	3.99	11.4	1.79	8.85	2.90	1.01	2.55	0.497	3.20	0.626	1.86	0.299	2.18	0.31	1.52	0.093	0.029	2.36	0.28	0.18
SHM-96-04	50.9	0.77	18.1	2.92	6.44	0.18	6.91	10.4	2.50	0.57	0.11	34	285	117	37	54	12	287	19	60	1.4	0.56	241	5.52	13.5	1.92	9.75	3.10	0.991	2.90	0.589	3.35	0.623	1.93	0.362	2.35	0.28	1.73	0.087	0.033	2.33	0.61	0.44
**Tolbachik**																																											
TOL-96-01	52.1	1.7	17.2	3.59	6.30	0.17	4.67	8.14	3.47	2.1	0.60	19	280		28	42	44	340	30	185	5.8	1.5	461	16.0	41.0	5.77	26.5	6.42	1.82	5.17	0.955	5.88	1.17	3.38	0.521	3.24	0.49	6.36	0.81	0.12	5.81	2.2	1.2
TOL-96-03	50.9	1.2	14.6	3.59	6.31	0.18	9.10	10.2	2.73	1.3	0.35	31	279		37	119	34	308	23	112	3.5	0.91	292	10.2	25.9	3.77	18.1	4.61	1.41	3.78	0.723	4.48	0.932	2.65	0.402	2.52	0.37	4.02	0.64	0.049	3.29	1.4	0.72
201	50.2	1.2	14.2	3.60	6.46	0.17	9.61	9.83	2.61	1.3	0.34	33	271	569	47	168	34	305	27	124	3.1	0.91	296	10.5	27.0	4.37	20.1	5.29	1.46	4.58	0.750	4.25	0.840	2.52	0.370	2.27	0.34	3.02	0.14	0.046	3.23	0.95	0.65
655	50.5	0.95	13.4	2.14	7.16	0.17	9.67	11.6	2.37	0.85	0.21	38	270	418	40	99	18	292	19	79	1.5	0.46	229	6.47	16.3	2.72	13.6	3.85	1.12	3.56	0.580	3.50	0.730	2.00	0.290	1.89	0.28	2.05	0.080	0.058	2.03	0.49	0.32
22-8	51.1	1.6	17.5	2.25	7.20	0.16	4.34	8.30	3.46	2.0	0.56	22	270	165	30	45	52	362	32	199	4.8	1.6	467	17.0	41.9	6.42	29.0	7.14	1.84	6.22	0.970	5.66	1.17	3.47	0.490	3.19	0.48	4.75	0.28	0.061	7.49	2.3	1.3
**Ichinsky**																																											
ICH-96-02	54.9	1.2	16.7	2.02	5.87	0.15	4.80	7.44	3.73	1.5	0.48	23	194	74	26	30	20	596	22	177	8.2	0.30	583	19.1	46.3	6.43	28.2	6.27	1.79	5.36	0.791	4.69	0.924	2.62	0.372	2.48	0.37	4.26	0.46	0.11	5.57	1.5	0.59
6250	52.0	0.85	14.7	3.01	5.62	0.16	8.41	10.1	2.66	1.5	0.23	32	243	346	39	92	25	503	18	95	3.7	0.38	443	9.03	22.2	3.30	15.2	3.82	1.11	3.31	0.520	3.10	0.610	1.73	0.260	1.67	0.25	2.50	0.42	0.12	4.25	1.5	0.71
replicate																																								0.12			
ICH-96-19	50.4	0.89	20.2	3.11	5.08	0.14	4.88	9.77	3.17	1.1	0.25	25	219	33	30	45	18	722	15	93	3.6	0.21	454	8.22	19.0	2.86	14.0	3.55	1.19	3.12	0.490	2.79	0.550	1.61	0.240	1.48	0.23	2.28	0.24	0.033	3.43	0.76	0.39
ICH-96-28	55.8	0.90	16.9	1.89	5.18	0.13	4.97	7.71	3.57	1.3	0.32	22	172	97	31	77	20	532	18	157	6.0	0.40	469	16.5	36.4	4.69	20.7	5.13	1.43	4.38	0.730	4.15	0.780	2.23	0.360	2.31	0.31	3.86	0.39	0.10	5.91	1.8	0.84
ICH-96-29	53.0	1.1	17.6	2.82	5.21	0.14	5.04	8.46	3.49	1.3	0.37	24	216	72	33	64	20	581	17	138	6.8	0.39	440	13.5	34.4	4.87	20.8	4.91	1.48	4.16	0.610	3.72	0.740	1.95	0.270	1.83	0.29	3.28	0.32	0.099	4.10	1.3	0.50
ICH-96-31	54.5	0.91	17.6	2.49	4.91	0.13	4.60	8.08	3.47	1.3	0.30	24	199	66	26	62	22	559	16	128	5.6	0.49	449	13.2	28.7	4.03	19.1	4.55	1.29	4.12	0.620	3.42	0.670	2.01	0.300	1.82	0.27	3.43	0.27	0.10	4.79	1.8	0.77

ICH-96-54	55.6	0.96	18.1	2.22	4.77	0.13	4.22	7.92	3.73	1.7	0.38	22	179	48	26	41	32	645	18	163	7.0	0.75	575	16.9	37.0	4.93	23.2	5.15	1.42	4.41	0.690	3.69	0.720	2.12	0.320	2.02	0.29	4.00	0.36	0.18	6.40	2.6	1.2
replicate																																								0.16			
ICH-96-05	47.4	1.9	16.8	3.87	6.44	0.17	7.38	9.83	3.57	1.2	0.47	27	262	129	45	68	17	690	19	145	17	0.20	317	18.9	43.4	5.82	26.0	6.42	1.99	5.31	0.824	4.68	0.832	2.18	0.310	1.99	0.29	3.49	0.84	0.043	2.17	1.6	0.58
ICH-96-10	47.8	1.8	16.4	3.77	6.52	0.17	8.64	8.51	3.43	1.4	0.51	24	235	187	46	184	19	686	20	147	15	0.29	403	17.0	38.4	5.34	25.3	5.58	1.79	5.02	0.733	4.07	0.792	2.20	0.297	1.83	0.27	3.44	0.55	0.018	2.18	1.6	0.57
ICH-96-69	50.5	1.5	16.8	5.41	4.17	0.17	6.94	8.92	3.69	1.3	0.59	28	245	163	40	113	19	709	23	164	14	0.34	501	20.3	46.7	6.45	29.4	6.64	1.91	5.47	0.808	4.61	0.884	2.46	0.359	2.32	0.35	3.85	0.68	0.020	4.04	1.5	0.58
replicate																																								0.021			
6283	49.5	1.6	16.7	9.52	0.890	0.16	6.76	8.75	3.76	1.4	0.49	27	241	247	38	94	19	672	24	152	13	0.24	469	17.0	42.9	5.94	26.2	6.07	1.74	5.10	0.780	4.40	0.850	2.36	0.340	2.13	0.32	3.54	0.92	0.018	4.62	1.4	0.50
replicate																																								0.018			
ICH-96-07	49.0	1.8	16.6	3.51	6.43	0.17	7.19	8.57	3.80	1.5	0.59	24	241	174	40	110	21	688	20	164	16	0.32	419	20.8	49.9	6.67	28.6	6.70	2.08	5.57	0.810	4.94	0.920	2.33	0.320	2.18	0.33	3.70	0.85	0.048	3.13	1.7	0.63
ICH-96-08	48.7	1.7	16.5	7.33	2.62	0.16	7.35	9.30	3.40	1.4	0.48	27	239	206	41	98	24	634	20	154	17	0.43	431	18.7	42.2	5.72	24.8	5.65	1.73	4.67	0.710	3.95	0.750	1.99	0.280	1.82	0.28	1.76	0.010	1.0	2.93	2.1	0.68
replicate																																								0.94			
ICH-96-13	52.7	1.5	17.3	2.46	5.80	0.15	4.52	7.37	4.02	2.2	0.50	20	195	47	26	36	37	616	22	208	22	0.71	549	20.7	45.0	6.39	27.8	5.58	1.68	5.23	0.700	4.15	0.900	2.53	0.330	2.08	0.33	4.79	1.0	0.034	2.54	3.2	1.1
**Esso**																																											
ESO-96-08	50.6	0.98	17.8	2.39	6.43	0.16	6.51	9.75	2.92	0.88	0.23	30	261	186	34	69	14	592	19	70	1.7	0.36	369	6.11	17.7	2.54	12.1	3.15	1.02	2.85	0.445	2.64	0.535	1.58	0.229	1.47	0.22	1.82	0.10	0.084	2.66	0.38	0.26
ESO-96-11	52.5	0.82	17.5	2.73	5.82	0.15	6.36	9.16	2.91	1.0	0.22	29	242	205	38	77	19	620	16	74	2.0	0.36	443	7.34	20.1	2.66	11.8	2.92	0.930	2.54	0.390	2.24	0.450	1.34	0.190	1.23	0.18	1.89	0.10	0.070	3.99	0.70	0.43
ESO-96-01	53.7	0.84	18.2	3.06	5.03	0.16	5.16	8.36	3.28	1.1	0.23	22	217	107	32	58	21	657	18	80	2.9	0.48	502	8.18	22.0	2.88	12.8	3.09	0.970	2.63	0.400	2.31	0.480	1.43	0.200	1.35	0.21	2.09	0.15	0.094	4.59	0.69	0.41
ESO-96-04	53.0	0.87	18.0	2.27	5.73	0.15	5.48	8.55	3.17	1.2	0.22	27	231	121	34	60	20	641	18	79	3.1	0.46	493	7.89	21.3	2.80	12.4	3.04	0.950	2.60	0.390	2.32	0.480	1.44	0.210	1.34	0.20	2.04	0.15	0.11	4.30	0.69	0.40
**Achtang**																																											
ACH-96-01	54.4	0.90	17.3	2.28	5.19	0.15	5.13	7.62	3.28	1.5	0.31	23	196	99	26	49	25	598	22	122	6.4	0.44	576	12.8	33.1	4.21	17.8	4.02	1.20	3.47	0.510	3.02	0.629	1.82	0.266	1.70	0.27	2.99	0.30	0.14	5.11	1.2	0.63
ACH-96-02	51.2	0.94	16.0	2.94	5.84	0.16	7.89	9.61	2.86	1.2	0.29	30	243	337	37	112	21	530	21	93	3.9	0.41	373	8.76	24.0	3.27	14.7	3.62	1.11	3.30	0.500	3.04	0.640	1.89	0.270	1.71	0.27	2.37	0.18	0.10	3.25	0.71	0.38
replicate																																								0.10			
ACH-96-03	53.4	0.98	17.0	2.31	5.77	0.15	6.15	8.19	3.19	1.3	0.28	22	216	163	31	90	21	583	21	115	5.5	0.36	461	10.6	28.5	3.72	16.1	3.77	1.14	3.34	0.470	2.87	0.610	1.79	0.240	1.52	0.26	2.74	0.23	0.11	4.03	1.0	0.47
**Kluchevskoy**																																											
KLU-96-03	53.7	0.85	15.3	2.06	6.42	0.16	8.88	9.40	2.89	0.76	0.16	36	240	426	37	151	12	324	15	81	1.2	0.42	290	5.41	13.6	2.17	10.4	2.94	0.904	2.89	0.451	2.87	0.607	1.68	0.259	1.61	0.25	1.88	0.089	0.051	3.06	0.61	0.41
KLU-96-12	54.0	1.1	18.1	2.89	5.61	0.16	5.03	8.14	3.60	1.2	0.22	21	259	31	33	31	19	397	22	104	2.0	0.51	434	8.43	21.1	3.39	15.8	4.14	1.27	3.77	0.631	3.84	0.816	2.36	0.336	2.15	0.34	2.55	0.14	0.078	3.39	0.80	0.49
KLU-96-15	53.4	0.86	14.6	3.71	4.94	0.17	8.67	10.0	2.74	0.91	0.17	38	246	431	40	110	16	333	18	75	1.3	0.44	307	6.80	17.0	2.71	12.3	3.36	1.02	3.04	0.509	3.05	0.657	1.84	0.278	1.69	0.26	1.89	0.088	0.034	2.79	0.71	0.39
**Bakening**																																											
BAK-95-17	51.2	1.2	16.3	2.01	6.52	0.16	8.29	8.90	3.38	1.0	0.29	29	236	349	38	130	21	529	23	108	4.7	0.64	346	11.0	26.7	4.04	18.6	4.65	1.39	4.05	0.653	3.82	0.749	2.17	0.316	2.04	0.31	2.73	0.29	0.075	2.81	1.4	0.60
BAK-95-24	49.7	1.0	16.2	2.46	6.91	0.17	9.83	9.67	2.87	0.70	0.21	34	261	494	46	169	15	423	21	82	2.8	0.49	241	7.59	19.4	2.99	13.8	3.66	1.15	3.36	0.542	3.29	0.668	1.88	0.274	1.81	0.26	2.08	0.33	0.039	2.28	0.89	0.42
replicate																																								0.040			
BAK-95-31	50.3	1.1	16.1	2.24	6.89	0.17	9.15	9.32	3.21	0.95	0.26	30	253	394	44	155	22	505	21	91	4.3	0.76	400	10.7	24.7	3.64	16.7	4.28	1.29	3.82	0.608	3.59	0.697	1.99	0.284	1.85	0.28	2.37	0.27	0.063	3.49	1.6	0.66
BAK-95-14	50.3	0.99	16.4	2.18	7.02	0.18	8.92	9.74	3.00	0.68	0.23	33	242	411	41	126	17	453	24	96	3.0	0.45	185	8.10	21.5	3.40	15.7	4.10	1.28	3.80	0.640	4.00	0.830	2.38	0.360	2.33	0.34	2.41	0.45	0.048	2.10	0.81	0.44
BAK-95-15	50.1	0.96	16.8	2.60	6.73	0.19	8.33	9.51	3.00	0.77	0.26	32	235	317	40	121	21	531	24	96	2.3	0.59	194	8.90	23.2	3.70	17.4	4.50	1.32	4.00	0.660	4.00	0.810	2.34	0.350	2.23	0.34	2.48	0.11	0.040	2.10	0.95	0.52
BAK-95-30	50.5	1.1	16.3	2.58	6.56	0.17	9.05	9.22	3.16	0.94	0.26	30	249	377	43	155	19	497	22	90	3.1	0.70	379	10.0	23.4	3.50	16.2	4.10	1.25	3.80	0.610	3.60	0.710	2.01	0.290	1.89	0.28	2.35	0.15	0.065	3.50	1.5	0.64
BAK-95-33	51.3	1.0	16.8	2.35	6.56	0.17	8.32	9.06	3.14	0.92	0.23	29	240	315	43	134	19	463	21	91	3.6	0.64	312	9.60	23.0	3.40	15.9	4.10	1.28	3.80	0.620	3.70	0.740	2.10	0.310	2.03	0.30	2.46	0.23	0.058	3.20	1.8	0.60
BAK-95-34	51.2	1.0	16.9	2.08	6.70	0.17	7.80	8.94	3.18	0.91	0.23	29	237	272	38	117	20	471	24	92	3.6	0.58	304	9.40	22.7	3.40	15.7	4.00	1.24	3.70	0.610	3.60	0.720	2.08	0.310	1.96	0.30	2.35	0.20	0.083	2.90	1.5	0.56
BAK-96-04	48.8	1.1	17.3	2.68	7.09	0.17	8.09	10.5	2.83	0.70	0.17	38	304	233	38	84	27	429	10	64	2.8	0.38	223	7.10	18.2	2.90	13.7	3.60	1.13	3.20	0.530	3.10	0.640	1.81	0.270	1.80	0.27	1.85	0.18	0.0088	2.10	0.67	0.33
replicate																																								0.0079			
0-96-26	50.4	1.1	16.3	2.53	6.42	0.15	9.40	9.06	3.22	0.90	0.28	30	250	343	43	167	17	547	20	92	3.5	0.29	340	10.1	23.8	3.60	16.6	4.10	1.28	3.70	0.590	3.40	0.660	1.91	0.280	1.79	0.26	2.38	0.22	0.028	2.80	1.2	0.55
23	49.7	1.2	15.8	2.81	6.79	0.16	9.30	9.33	3.05	1.4	0.39	28	243	545	43	230	26	558	21	117	7.5	0.60	329	12.5	30.8	4.60	20.9	5.10	1.49	4.20	0.640	3.60	0.670	1.84	0.260	1.66	0.26	2.64	0.48	0.043	1.80	1.3	0.68
replicate																																								0.040			
23/1	49.8	1.2	15.8	4.30	5.35	0.16	9.17	9.33	3.08	1.4	0.39	30	243	539	47	227	28	554	20	119	7.5	0.43	314	12.7	31.1	4.70	21.2	5.20	1.48	4.30	0.640	3.60	0.680	1.88	0.260	1.69	0.25	2.62	0.49	0.012	1.50	1.3	0.69
replicate																																								0.010			
**Nikolka**																																											

8868	55.2	1.3	17.8	4.42	4.12	0.14	2.46	7.18	3.48	2.2	0.33	22	282	202	21	10	46	419	27	142	3.0	2.0	743	14.4	35.7	5.28	24.7	6.33	1.65	5.41	0.880	3.14	0.980	2.85	0.410	2.64	0.39	4.02	0.49	0.081	6.50	1.7	1.2
8878	56.0	0.85	16.7	1.78	5.70	0.14	4.19	7.40	3.30	1.2	0.17	29	227	330	24	1.0	19	416	18	93	1.7	0.24	478	8.12	19.4	2.98	14.2	3.67	1.12	3.26	0.540	5.33	0.640	1.83	0.280	1.71	0.25	2.51	0.34	0.20	3.72	0.65	0.51
replicate																																								0.17			
8883	51.1	1.4	18.4	4.79	4.96	0.17	3.56	8.20	3.56	1.4	0.61	25	265	148	28	22	41	469	30	176	4.4	0.23	689	20.6	51.3	7.66	33.9	7.65	1.93	6.16	0.940	2.87	1.07	3.05	0.440	2.86	0.43	4.38	0.50	0.049	7.19	2.5	1.4
replicate																																								0.045			

[a]Major elements in wt. % and trace elements (including thallium) in μg/g. All data (except thallium) are from refs.[6,15]. Thallium data are from this study.

[b]Replicate denotes repeated sample dissolution, column chemistry and instrumental analyses.

Supplementary Table 2 Sr, Nd, Pb and Tl isotopes for lavas from Kamchatka arc[a]

Sample	$^{87}Sr/^{86}Sr$	$^{143}Nd/^{144}Nd$	$^{206}Pb/^{204}Pb$	$^{207}Pb/^{204}Pb$	$^{208}Pb/^{204}Pb$	$\varepsilon^{205}Tl$	2sd[b]	n[c]
**Gamchen**								
GAM-96-14	0.703401	0.512991	18.316	15.508	38.138	***+13.5***	0.4	2
GAM-96-16						***-0.3***	0.4	2
GAM-96-28	0.703346	0.513033	18.335	15.510	38.070	***+1.2***	0.4	2
GAM-96-07						***-0.9***	0.4	2
GAM-96-12	0.703401	0.512991	18.316	15.508	38.138	***+0.6***	0.4	2
GAM-96-22						**-2.1**	0.4	2
GAM-96-26						**-1.5**	0.4	2
**Kizimen**								
KIZ-96-01						**-2.1**	0.4	2
KIZ-96-19						**-4.7**	0.4	2
KIZ-96-24	0.703347	0.513048				**-1.0**	0.4	2
KIZ-96-01/1	0.703352	0.513045				**-4.1**	0.4	2
KIZ-96-24/1	0.703370	0.513047	18.320	15.502	38.033	**-0.9**	0.4	2
TAM-96-01						***+11.5***	0.4	2
replicate[d]						***+11.9***	0.4	2
**Komarov**								
KOM-96-02/2	0.703482	0.513036	18.301	15.495	38.037	**-2.5**	0.4	2
KOM-96-06	0.703386	0.513044	18.343	15.528	38.159	***-0.6***	0.4	2
KOM-96-01						***-0.8***	0.4	2
KOM-96-14						***+0.7***	0.4	2
**Shmidt**								
SHM-96-01	0.703344	0.513070	18.306	15.495	37.960	***+8.6***	0.4	2
SHM-96-03						**+3.6**	0.4	2
SHM-96-04	0.703383	0.513032				**+0.9**	0.4	2
**Tolbachik**								
TOL-96-01	0.703374	0.513141				**+1.1**	0.4	2
TOL-96-03	0.703356	0.513081	18.185	15.472	37.850	**-0.1**	0.4	2
201	0.703341	0.513095	18.192	15.482	37.885	**+0.5**	0.4	2
655	0.703368	0.513077				**-0.8**	0.4	2
22-8	0.703331	0.513106				**-1.1**	0.4	2
**Ichinsky**								
ICH-96-02	0.703379	0.513046				**-1.7**	0.4	2
6250	0.703344	0.513077				**-2.0**	0.4	2
replicate						**-1.7**	0.4	2
ICH-96-19	0.703329	0.513054	18.248	15.497	37.953	**-0.2**	0.4	2
ICH-96-28						**-1.5**	0.4	2
ICH-96-29						**-0.8**	0.4	2
ICH-96-31	0.703364	0.513041	18.248	15.488	37.975	**-1.0**	0.4	2
ICH-96-54						**-1.3**	0.4	2
replicate						**-1.6**	0.4	2
ICH-96-05	0.703395	0.512987	18.057	15.476	37.952	**0.0**	0.4	2
ICH-96-10	0.703405	0.512974	18.092	15.462	37.896	**+1.4**	0.4	2
ICH-96-69	0.703301	0.513021	18.192	15.473	37.902	**-0.3**	0.4	2
replicate						**-0.2**	0.4	2
6283	0.703352	0.513035				**-1.9**	0.4	2
replicate						**-1.6**	0.4	2
ICH-96-07	0.703391	0.512997				**-1.2**	0.4	2
ICH-96-08						**-4.5**	0.4	2
replicate						**-4.7**	0.4	2
ICH-96-13						**-1.4**	0.4	2
**Esso**								
ESO-96-08	0.703355	0.513092	18.249	15.486	37.916	**-1.7**	0.4	2
ESO-96-11						***-0.2***	0.4	2
ESO-96-01						***-1.1***	0.4	2
ESO-96-04	0.703342	0.513063				**-1.5**	0.4	2
**Achtang**								
ACH-96-01	0.703354	0.513039	18.199	15.460	37.841	**-1.8**	0.4	2
ACH-96-02	0.703352	0.513044				**-1.7**	0.4	2
replicate						**-2.1**	0.4	2
ACH-96-03	0.703295	0.513032				**-1.8**	0.4	2
**Kluchevskoy**								
KLU-96-03	0.703585	0.513080	18.281	15.500	37.971	**-0.6**	0.4	2
KLU-96-12	0.703664	0.513102	18.303	15.509	37.997	**-0.5**	0.4	2

KLU-96-15	0.703509	0.513109	18.300	15.489	37.941	***+2.4***	0.4	2
**Bakening**								
BAK-95-17	0.703150	0.513070	18.298	15.493	37.998	**-0.7**	0.4	2
BAK-95-24	0.703170	0.513060	18.345	15.501	38.062	***+1.7***	0.4	2
replicate						***+1.4***	0.4	2
BAK-95-31	0.703290	0.513030	18.334	15.482	38.032	***+3.4***	0.4	2
BAK-95-14						***+0.4***	0.4	2
BAK-95-15						***+1.2***	0.4	2
BAK-95-30	0.703270	0.513040				***+0.5***	0.4	2
BAK-95-33						***+0.9***	0.4	2
BAK-95-34						**-0.4**	0.4	2
BAK-96-04	0.703110	0.513070	18.347	15.492	38.015	***+6.7***	0.4	2
replicate						***+6.9***	0.4	2
O-95-26	0.703220	0.513070				***+4.7***	0.4	2
23						***+3.9***	0.4	2
replicate						***+3.8***	0.4	2
23/1						***+8.3***	0.4	2
replicate						***+8.9***	0.4	2
**Nikolka**								
8868	**0.703703**	**0.513113**	**18.266**	**15.480**	**37.898**	**-1.1**	0.4	2
8878	**0.703597**	**0.513085**	**18.294**	**15.488**	**37.949**	**-0.9**	0.4	2
replicate						**-1.0**	0.4	2
8883	0.703441	0.513097				**+0.5**	0.4	2
replicate						**+0.3**	0.4	2

[a]Most Sr, Nd and Pb isotope data are from refs.[6,15]. Bold data of Sr, Nd and Pb isotopes and all thallium isotope data are from this study. Italic Tl isotope data represent samples affected by magma degassing.

[b]We analyzed two times each sample using mass spectrometry for Tl isotope data. Although individual Tl isotope analyses have uncertainties of 0.01-0.37 (2sd), we apply the external 2sd reproducibility of 0.4 ε^{205}Tl-units to all unknowns because this uncertainty accounts for all possible sources of error including sample dissolution, ion exchange chromatography and mass spectrometric procedures.

[c]n represents the numbers of mass spectrometry analyses for the same sample.

[d]Replicate denotes repeated sample dissolution, column chemistry and instrumental analyses.

Supplementary Table 3 Lithology, unit and density of samples selected from DSDP Expedition 19 Site 192 and ODP Expedition 145 Hole 881C.

Sample (Site-Core-Section)	Interval (cm)	Depth (m sub-bottom)	Depth (m sub-basement)	Lithology	Unit	Unit density (g/cm^3)	Unit thickness (m)
**Sediment from the Hawaii-Emperor Seamount Chain**							
**DSDP Site 192**							
192 2-3	85-90	4.85		Diatom rich silty clay			
192 4-4	100-105	24.50		Diatomaceous silty clay	A-1	1.6	140
192 8-5	80-85	98.80		Diatomaceous silty clay			
192 13-4	70-75	237.20		Silt bearing clay rich diatom ooze			
192 19-1	60-65	391.60		Silt rich clayey diatom ooze	A-2	1.5	410
192 22-4	70-75	527.20		Silt bearing clay rich diatom ooze			
192 23-2	70-75	571.20		Silt bearing diatom rich clay			
192 24-2	90-95	627.40		Silt bearing diatom rich clay	A-3	1.5	155
192 25-2	55-60	673.05		Clayey diatom ooze			
192 27-3	90-95	749.90		Silty claystone			
192 30-1	110-115	850.10		Silty claystone	B	1.9	235
192 34-4	75-80	927.25		Claystone			
192A 1-3	115-120	946.15		Nannofossil chalk			
192A 2-4	105-110	956.55		Claystone	C	2.1	104
192A 4-4	60-65	1023.10		Claystone			
**Pelagic sediment**							
**ODP Hole 881C**							
881C 1-1	70-75	0.70		Diatom ooze and clay			
881C 6-3	60-65	46.80		Diatom ooze and clay			
881C 10-5	75-80	88.05		Diatom ooze and clay	IA	1.45	164.5
881C 12-2	80-85	102.70		Diatom ooze and clay			
881C 17-3	90-95	151.65		Diatom ooze and clay			
881C 21-2	45-50	185.15		Diatom ooze			
881C 25-3	60-65	223.60		Diatom ooze			
881C 30-3	80-85	272.50		Diatom ooze	IB	1.25	199.3
881C 35-3	70-75	320.55		Diatom ooze			
881C 36-4	70-75	331.80		Diatom ooze			
**Basalt from the Hawaii-Emperor Seamount Chain**							
**DSDP Site 192**							
192A 5-2	35-40	1045.35	1.35	Pyroxene-plagioclase diabasic basalt			
192A 5-4	115-120	1049.15	5.15	Pyroxene-plagioclase diabasic basalt			
192A 5-6	115-120	1052.15	8.15	Pyroxene-plagioclase diabasic basalt			
192A 6-2	50-55	1054.00	10.00	Pyroxene-plagioclase diabasic basalt			
192A 6-3	50-55	1055.50	11.50	Pyroxene-plagioclase diabasic basalt			

112 Supplementary Table 4 Major and trace element compositions of sediments and Hawaii-Emperor Seamount Chain outboard of the Kamchatka arc[a]

Sample	SiO_2	TiO_2	Al_2O_3	FeO*	MnO	MgO	CaO	Na_2O	K_2O	P_2O_5	LOI	Li	Be	Sc	V	Co	Ni	Rb	Sr	Y	Nb	Mo	Cd	Cs	Ba	La	Ce	Pr	Nd	Sm	Eu	Gd	Tb	Dy	Ho	Er	Tm	Yb	Lu	Tl	Pb	Th	U
**Sediment from the Hawaii-Emperor Seamount Chain**																																											
**DSDP Site 192**																																											
192 2-3	57.9	0.61	13.4	5.39	0.14	2.53	3.60	3.75	2.04	0.13	8.60	43.0	1.45	18.8	144	76.5	41.9	55.5	312	20.4	5.63	1.2	0.27	3.39	1416	15.5	34.6	4.21	16.8	3.74	0.994	3.73	0.59	3.53	0.73	2.15	0.32	2.11	0.31	0.24	17.5	4.65	1.76
replicate[b]																																								0.26			
192 4-4	60.6	0.65	13.2	5.39	0.13	2.39	2.07	3.38	2.28	0.12	8.09	54.3	1.76	17.9	151	58.4	46.4	78.8	224	20.3	8.26	0.65	0.18	4.96	1376	20.8	44.4	5.10	19.5	4.07	0.993	3.87	0.60	3.55	0.73	2.13	0.32	2.07	0.31	0.30	19.5	6.68	1.81
192 8-5	59.5	0.65	13.0	5.95	0.18	2.73	1.58	3.41	2.20	0.11	9.00	55.3	1.56	18.5	180	51.3	43.1	72.7	198	18.7	7.12	0.96	0.96	4.58	1469	16.6	37.7	4.25	16.6	3.61	0.930	3.57	0.55	3.31	0.69	2.00	0.30	2.07	0.29	0.33	17.4	5.35	2.23
192 13-4	70.3	0.31	5.82	2.58	0.041	1.16	2.02	2.90	0.928	0.047	11.5	27.9	0.783	9.62	83.0	106	24.1	25.1	154	9.00	2.50	0.59	1.3	1.63	213	6.55	16.1	1.79	7.09	1.61	0.429	1.62	0.25	1.52	0.32	0.956	0.15	1.02	0.16	0.16	6.48	1.98	4.10
192 19-1	59.9	0.55	11.1	5.16	0.070	2.36	1.23	3.55	1.85	0.078	11.8	44.6	1.11	13.7	121	79.8	46.0	46.3	155	12.8	4.55	0.97	0.95	3.11	119	10.3	22.9	2.66	10.6	2.36	0.622	2.41	0.38	2.28	0.47	1.40	0.21	1.42	0.21		10.4	3.17	1.97
192 22-4	58.2	0.44	9.26	4.14	0.17	2.02	2.23	4.23	1.50	0.098	14.6	50.0	1.26	13.6	120	42.3	48.9	42.9	177	13.6	4.59	1.0	1.1	2.61	98.5	11.6	25.0	3.04	12.2	2.70	0.687	2.65	0.41	2.45	0.51	1.47	0.22	1.45	0.22	0.31	9.25	3.28	2.39
192 23-2	60.1	0.53	11.1	4.94	0.15	2.24	1.99	3.28	1.78	0.070	11.6	63.1	1.49	15.8	137	38.9	54.2	54.8	215	19.1	5.86	0.42	0.26	3.40	382	16.1	31.8	4.15	16.4	3.62	0.930	3.66	0.57	3.40	0.70	2.00	0.30	1.93	0.28	0.18	14.0	4.19	1.50
192 24-2	60.6	0.61	13.2	5.62	0.080	2.33	1.21	3.31	2.09	0.078	9.48	64.5	1.57	17.5	148	31.0	45.0	65.2	211	17.4	6.93	0.50	0.14	3.85	576	16.6	34.4	4.35	17.0	3.64	0.931	3.49	0.54	3.15	0.64	1.85	0.27	1.81	0.26	0.21	14.1	4.92	1.65
192 25-2	63.8	0.45	9.54	3.96	0.11	1.73	2.85	2.76	1.53	0.062	11.1	49.9	1.36	13.7	100	26.3	39.5	49.8	236	15.1	5.20	0.58	0.19	3.11	490	12.3	24.2	3.14	12.4	2.72	0.711	2.76	0.43	2.63	0.56	1.64	0.25	1.67	0.25	0.18	10.3	3.84	1.33
192 27-3	60.2	0.69	14.3	6.68	0.095	2.66	1.46	2.81	2.19	0.079	7.90	84.9	2.17	21.0	157	25.1	50.5	73.7	296	18.6	7.50	0.54	0.12	4.64	2504	18.3	36.7	4.84	19.0	4.16	1.12	4.03	0.62	3.57	0.72	2.05	0.30	1.99	0.29	0.21	15.0	5.09	1.66
replicate																																								0.20			
192 30-1	63.2	0.66	14.3	6.08	0.072	2.38	0.963	2.23	2.33	0.084	6.57	61.9	2.36	20.3	163	68.5	37.6	77.5	240	17.9	8.10	0.31	0.071	4.48	2620	16.8	37.5	4.23	16.1	3.46	0.904	3.31	0.52	3.13	0.65	1.95	0.30	2.11	0.32	0.24	13.0	5.66	1.82
192 34-4	62.5	0.60	12.5	6.32	0.28	3.14	1.83	2.39	1.89	0.14	6.93	76.4	2.82	25.5	101	40.9	85.8	46.6	447	45.6	4.81	0.20	0.042	2.88	4104	19.4	39.0	6.15	25.2	6.26	1.68	6.41	1.0	6.27	1.3	4.02	0.65	4.77	0.78	0.15	19.4	3.46	0.891
192A 1-3	25.2	0.11	2.24	1.21	0.45	0.575	36.9	0.518	0.403	0.035	31.0	22.5	0.596	6.00	33.0	63.5	25.4	10.2	1090	13.1	0.460	0.081	0.55	0.596	1720	10.5	10.2	3.21	13.2	2.94	0.751	2.90	0.42	2.39	0.45	1.21	0.17	1.05	0.15	0.062	2.11	0.630	0.350
192A 2-4	48.5	0.57	9.28	6.03	0.30	2.91	12.4	2.08	1.16	0.13	15.2	58.8	1.18	22.8	129	29.1	62.7	21.5	733	28.0	1.56	0.15	0.18	0.682	4893	19.3	27.4	6.63	27.9	6.69	1.79	6.57	1.0	5.67	1.1	2.94	0.42	2.71	0.39	0.057	9.39	0.831	0.222
192A 4-4	21.0	0.36	5.72	3.48	0.23	2.49	32.9	0.584	2.16	0.14	29.3	42.5	0.790	15.3	36.3	19.1	65.2	20.3	687	30.1	1.74	0.17	0.66	0.834	2479	19.1	25.5	5.81	24.5	5.76	1.50	5.93	0.90	5.20	1.0	2.81	0.39	2.49	0.35	0.25	7.99	1.40	0.210
**Pelagic sediment**																																											
**ODP Hole 881C**																																											
881C 1-1	57.4	0.65	13.5	5.66	0.12	2.51	1.91	3.81	2.26	0.12	9.85	139	1.66	17.9	161	42.2	38.4	79.9	196	19.9	8.48	0.75	0.14	5.38	997	20.6	46.1	5.07	19.1	4.03	0.973	3.87	0.60	3.57	0.74	2.16	0.32	2.13	0.31	0.33	20.5	7.45	1.81
replicate												140	1.69	18.2	162	42.4	38.5	79.2	195	19.9	8.38	0.72	0.14	5.38	990	20.6	45.5	5.04	19.3	4.03	0.966	3.85	0.60	3.53	0.74	2.16	0.32	2.11	0.31		20.4	7.49	1.92
881C 6-3	58.9	0.66	13.9	5.47	0.10	2.23	2.07	3.85	2.30	0.11	8.73	55.4	1.78	20.6	152	35.9	32.7	77.1	202	22.6	7.34	1.9	0.17	5.17	1137	19.8	45.8	4.98	19.5	4.21	1.04	4.09	0.66	3.93	0.82	2.38	0.35	2.44	0.34	0.34	19.6	6.63	2.00
881C 10-5	58.4	0.69	14.3	6.06	0.11	2.32	2.37	3.62	2.36	0.13	7.86	48.9	1.74	22.1	146	41.1	29.2	80.8	219	23.3	7.32	1.5	0.16	5.47	931	19.3	43.8	4.97	19.5	4.24	1.06	4.17	0.67	4.00	0.84	2.44	0.36	2.39	0.35	0.32	19.5	6.82	1.50
881C 12-2	59.2	0.56	12.6	5.24	0.10	2.09	2.20	4.18	1.86	0.085	9.60	38.7	1.38	21.3	117	50.9	29.2	54.7	194	22.3	4.70	0.76	0.17	3.86	1216	13.7	35.3	3.85	15.5	3.72	0.990	3.88	0.64	3.94	0.84	2.45	0.37	2.43	0.36	0.19	20.8	4.56	1.04
881C 17-3	61.8	0.50	12.3	4.60	0.11	2.20	1.42	3.67	2.35	0.073	9.16	43.3	1.69	17.8	109	38.2	40.7	67.1	156	20.2	5.79	0.88	0.088	4.64	893	17.6	43.8	4.61	17.8	3.93	0.912	3.80	0.60	3.60	0.74	2.16	0.32	2.13	0.31	0.25	19.8	6.19	1.54
881C 21-2	76.2	0.14	3.13	1.47	0.072	0.864	0.419	2.86	0.688	0.020	12.3	16.0	0.652	5.99	29.5	108	22.7	20.9	60.7	5.86	1.74	0.46	0.040	1.21	874	6.13	16.4	1.67	6.53	1.47	0.369	1.42	0.22	1.23	0.24	0.672	0.10	0.646	0.10	0.072	11.1	2.02	0.545
881C 25-3	78.4	0.060	1.31	0.620	0.083	0.782	0.253	2.68	0.368	0.0083	13.1	11.2	0.347	2.81	20.9	166	36.2	9.70	34.2	3.09	0.482	2.6	0.33	0.789	405	3.28	8.19	0.857	3.40	0.751	0.180	0.739	0.11	0.638	0.13	0.353	0.052	0.335	0.049	0.10	102	0.855	0.632
replicate																																								0.097			
881C 30-3	73.0	0.20	4.51	1.86	0.067	1.09	0.446	2.82	0.963	0.022	12.3	22.9	1.19	9.81	47.1	83.2	69.3	31.8	67.7	8.48	2.67	0.99	0.49	2.36	335	10.8	28.8	2.96	11.9	2.67	0.639	2.50	0.37	2.05	0.39	1.06	0.15	1.01	0.14	0.20	12.9	3.27	1.19
881C 35-3	70.8	0.26	6.25	2.88	0.21	1.28	0.485	2.75	1.27	0.026	11.7	24.1	1.62	12.8	51.2	86.8	54.2	47.6	81.1	9.68	3.78	0.48	0.10	3.54	851	13.2	39.5	3.65	13.8	3.01	0.710	2.72	0.41	2.23	0.42	1.16	0.17	1.11	0.16	0.17	18.8	4.95	0.837
881C 36-4	73.7	0.20	4.70	2.14	0.18	0.934	0.436	2.62	0.980	0.030	11.9	15.1	1.26	9.39	34.8	96.1	37.2	39.0	69.0	7.97	3.03	0.72	0.054	2.89	1091	10.3	32.5	2.89	10.8	2.36	0.569	2.15	0.33	1.92	0.34	0.945	0.14	0.898	0.13	0.16	17.1	3.88	0.745
**Basalt from the Hawaii-Emperor Seamount Chain**																																											
**DSDP Site 192**																																											
192A 5-2	47.5	1.8	14.9	11.1	0.18	4.21	12.1	2.64	1.34	0.20	3.33	13.6	0.710	41.2	342	100	37.5	19.9	202	27.6	8.05	0.50	0.23	0.203	48.3	7.36	19.6	2.77	12.9	3.64	1.30	4.35	0.73	4.59	0.96	2.72	0.39	2.47	0.36	0.037	0.753	0.562	0.279
192A 5-4	48.6	1.8	16.3	9.01	0.14	5.60	9.07	2.72	1.83	0.20	4.49	40.6	0.643	41.1	297	72.0	37.2	12.5	218	31.3	9.04	0.42	0.17	0.102	77.8	8.33	21.7	3.15	14.2	4.00	1.43	4.80	0.80	4.99	1.0	2.98	0.42	2.69	0.38	0.061	0.734	0.611	0.611
192A 5-6	46.7	1.8	14.0	10.8	0.15	4.84	11.1	2.38	2.14	0.18	5.11	22.6	0.680	46.1	335	62.2	34.0	36.3	210	29.7	8.02	0.38	0.19	0.464	60.1	7.92	21.0	2.92	13.6	3.81	1.38	4.70	0.79	4.94	1.1	3.00	0.43	2.74	0.40	0.061	0.269	0.556	0.727
192A 6-2	47.0	2.0	14.4	9.50	0.16	5.13	11.8	2.71	1.22	0.22	5.13	20.3	0.596	45.7	359	86.4	48.6	17.9	221	29.9	8.87	0.34	0.50	0.439	54.3	8.33	21.8	3.18	14.7	4.14	1.47	4.90	0.83	5.11	1.1	3.01	0.43	2.71	0.38	0.026	0.729	0.631	0.449
192A 6-3	48.2	2.0	14.5	10.8	0.18	5.63	11.4	2.78	0.640	0.21	3.10	21.0	0.583	47.3	364	120	71.3	5.30	218	32.1	9.00	0.42	0.25	0.108	49.6	8.30	21.9	3.19	14.8	4.24	1.49	5.07	0.85	5.28	1.1	3.13	0.44	2.80	0.40	0.012	0.699	0.624	0.292
replicate																																								0.011			

[a]Major elements (including LOI) in wt. % and trace elements (including Tl) in μg/g; FeO*, total Fe.
[b]Replicate denotes repeated sample dissolution, column chemistry and instrumental analyses.

Supplementary Table 5 Sr, Nd, Pb and Tl isotopes for sediments and Hawaii-Emperor Seamount Chain outboard of the Kamchatka arc

Sample	$^{87}Sr/^{86}Sr$	$^{143}Nd/^{144}Nd$	$^{206}Pb/^{204}Pb$	$^{207}Pb/^{204}Pb$	$^{208}Pb/^{204}Pb$	$\varepsilon^{205}Tl$	2sd[a]	n[b]
**Sediment from the Hawaii-Emperor Seamount Chain**								
**DSDP Site 192**								
192 2-3						-1.4	0.4	2
replicate[c]						-1.6	0.4	2
192 4-4	0.707912	0.512464	18.894	15.612	38.703	-1.9	0.4	2
192 8-5						-1.9	0.4	2
192 13-4						-2.2	0.4	2
192 19-1	0.706630	0.512629	18.853	15.613	38.641			
192 22-4						-1.3	0.4	2
192 23-2						-2.3	0.4	2
192 24-2	0.706579	0.512608	18.820	15.604	38.627	-2.5	0.4	2
192 25-2						-2.1	0.4	2
192 27-3						-2.4	0.4	2
replicate						-2.5	0.4	2
192 30-1	0.707324	0.512583	18.851	15.608	38.655	-2.7	0.4	2
192 34-4						-1.7	0.4	2
192A 1-3						+0.3	0.4	2
192A 2-4	0.707129	0.512545	18.487	15.571	38.399	+3.4	0.4	2
192A 4-4						-4.7	0.4	2
**Pelagic sediment**								
**ODP Hole 881C**								
881C 1-1						-1.9	0.4	2
881C 6-3						-1.8	0.4	2
881C 10-5						-2.0	0.4	2
881C 12-2						-2.1	0.4	2
881C 17-3						-1.8	0.4	2
881C 21-2						-2.5	0.4	2
881C 25-3						+0.9	0.4	2
replicate						+1.0	0.4	2
881C 30-3						-0.3	0.4	2
881C 35-3						-1.2	0.4	2
881C 36-4						-0.1	0.4	2
**Basalt from the Hawaii-Emperor Seamount Chain**								
**DSDP Site 192**								
192A 5-2	0.703653	0.513019	18.559	15.473	38.042	+5.0	0.4	2
192A 5-4	0.704027	0.513013	18.813	15.479	38.009	+4.2	0.4	2
192A 5-6	0.704473	0.513043	21.099	15.602	38.371	+2.1	0.4	2
replicate			21.095	15.601	38.376			
192A 6-2	0.703921	0.513024	18.725	15.481	38.084	+0.9	0.4	2
192A 6-3	0.703623	0.513030	18.651	15.473	38.094	+1.0	0.4	2
replicate						+0.9	0.4	2

[a]We analyzed two times each sample using mass spectrometry for Tl isotope data. Although individual Tl isotope analyses have uncertainties of 0.01-0.28 (2sd), we apply the external 2sd reproducibility of 0.4 ε^{205}Tl-units to all unknowns because this uncertainty accounts for all possible sources of error including sample dissolution, ion exchange chromatography and mass spectrometric procedures.

[b]n represents the numbers of mass spectrometry analyses for the same sample.

[c]Replicate denotes repeated sample dissolution, column chemistry and instrumental analyses.

Supplementary Table 6 Weighted average compositions for sediments and Hawaii-Emperor Seamount Chain basalts outboard of the Kamchatka arc[a]

	Sediment from the Hawaii-Emperor Seamount Chain (DSDP Site 192)	Pelagic sediment outboard of Kamchatka arc (ODP Hole 881C)	Basalt from the Hawaii-Emperor Seamount Chain （DSDP Site 192）
SiO_2	58.0	67.0	47.6
TiO_2	0.52	0.39	1.9
Al_2O_3	10.6	8.55	14.8
FeO*	4.85	3.56	10.2
MnO	0.15	0.12	0.16
MgO	2.22	1.62	5.08
CaO	5.04	1.18	11.1
Na_2O	2.90	3.27	2.65
K_2O	1.73	1.53	1.43
P_2O_5	0.09	0.06	0.2
LOI	12.0	10.7	4.23
Li	53.3	40.9	23.6
Be	1.51	1.33	0.640
Sc	16.4	13.9	44.3
V	120	85.7	339
Co	55.4	75.6	88.1
Ni	47.2	39.1	45.7
Rb	49.1	50.4	18.4
Sr	308	127	214
Y	19.1	14.2	30.1
Nb	4.99	4.49	8.60
Mo	0.60	1.1	0.41
Cd	0.56	0.17	0.27
Cs	3.0	3.5	0.26
Ba	1471	869	58.0
La	14.4	13.4	8.04
Ce	29.0	33.8	21.2
Pr	3.95	3.53	3.04
Nd	15.8	13.7	14.0
Sm	3.56	3.02	3.97
Eu	0.93	0.74	1.4
Gd	3.54	2.91	4.76
Tb	0.55	0.46	0.80
Dy	3.26	2.69	4.98
Ho	0.67	0.54	1.1
Er	1.95	1.56	2.97
Tm	0.29	0.23	0.42
Yb	1.99	1.54	2.68
Lu	0.30	0.22	0.38
Tl	0.21	0.21	0.039
Pb	12.0	26.3	0.640
Th	3.63	4.63	0.597
U	1.86	1.17	0.470
$^{87}Sr/^{86}Sr$	0.707030		0.703940
$^{143}Nd/^{144}Nd$	0.512583		0.513026
$^{206}Pb/^{204}Pb$	18.807		18.890
$^{207}Pb/^{204}Pb$	15.605		15.487
$^{208}Pb/^{204}Pb$	38.620		38.083
$\varepsilon^{205}Tl$	-2.0	-1.2	+3.1

[a]The weighted average sediment compositions of the sediment from the Hawaii-Emperor Seamount Chain and pelagic sediment outboard of Kamchatka arc were calculated using the concentration and isotope data obtained for individual samples and combining them with the thicknesses and densities of the different sediment units present in all the drill cores (Supplementary Table 3). The weighted average compositions of the ridge lavas from the Hawaii-Emperor Seamount Chain were based on assigning each sample the same weight as the drill cores do not have sufficient stratigraphic information to weight the samples by lithological abundance. Major elements (including LOI) in wt. % and trace elements (including Tl) in μg/g.

Supplementary Table 7 Major and trace element abundances and Sr, Nd, Pb and Tl isotope compositions in filtered samples (see discussion) from Kamchatka arc[a]

	**Gamchen**		**Kizimen**					**Komarov**	**Shmidt**		**Tolbachik**					**Ichinsky**
	GAM-96-22	GAM-96-26	KIZ-96-01	KIZ-96-19	KIZ-96-24	KIZ-96-01/1	KIZ-96-24/1	KOM-96-02/2	SHM-96-03	SHM-96-04	TOL-96-01	TOL-96-03	201	655	22-8	ICH-96-02
	EVF	EVF	EVF	EVF	EVF	EVF	EVF	EVF	EVF	EVF	CKD	CKD	CKD	CKD	CKD	SR
SiO_2	52.8	50.6	63.6	50.3	54.7	49.7	51.6	53.7	59.1	50.9	52.1	50.9	50.2	50.5	51.1	54.9
TiO_2	0.80	1.0	0.58	1.2	1.0	1.2	1.3	0.75	0.70	0.77	1.7	1.2	1.2	0.95	1.6	1.2
Al_2O_3	17.2	18.5	16.2	16.5	17.4	18.8	17.9	16.0	18.4	18.1	17.2	14.6	14.2	13.4	17.5	16.7
Fe_2O_3	2.49	2.87	2.41	2.97	3.07	5.32	4.29	1.86	3.78	2.92	3.59	3.59	3.60	2.14	2.25	2.02
FeO	6.88	8.48	3.03	8.01	5.49	5.56	5.78	6.48	3.49	6.44	6.30	6.31	6.46	7.16	7.20	5.87
MnO	0.19	0.20	0.13	0.21	0.17	0.19	0.19	0.17	0.21	0.18	0.17	0.18	0.17	0.17	0.16	0.15
MgO	5.95	4.94	2.44	5.22	4.16	5.20	4.42	7.33	2.21	6.91	4.67	9.10	9.61	9.67	4.34	4.80
CaO	9.54	9.93	5.34	9.59	8.18	9.25	9.13	9.36	6.99	10.4	8.14	10.2	9.83	11.6	8.30	7.44
Na_2O	2.78	2.60	3.69	2.70	3.18	2.74	3.03	2.55	3.60	2.50	3.47	2.73	2.61	2.37	3.46	3.73
K_2O	0.54	0.61	1.7	0.71	1.1	0.76	0.87	0.80	0.46	0.57	2.1	1.3	1.3	0.85	2.0	1.5
P_2O_5	0.12	0.11	0.16	0.21	0.17	0.17	0.19	0.12	0.060	0.11	0.60	0.35	0.34	0.21	0.56	0.48
LOI	0.60	0.46	0.61	1.9	0.47	0.73	0.85	0.70	0.96	0.55	0.43	0.36	0.47	0.63	0.79	0.60
Sc	34	35	15	35	22	26	33	35	18	34	19	31	33	38	22	23
V	269	359	114	316	246	300	324	246	34.0	285	280	279	271	270	270	194
Cr	87.0	26.0	17.0	42.0	24.0	15.0	21.0	309		117			569	418	165	74.0
Co	35	38	14	37	27	30	27	33	7.0	37	28	37	47	40	30	26
Ni	46.0	13.0		25.0	2.00	2.00		89.0		54.0	42.2	119	168	99.0	45.0	30.0
Rb	8.0	9.0	38	9.3	21	14	16	15	7.3	12	44	34	34	18	52	20
Sr	257	268	319	276	328	370	335	219	408	287	340	308	305	292	362	596
Y	19	19	16	32	20	21	23	27	17	19	30	23	27	19	32	22
Zr	73.0	61.0	121	104	99.0	86.0	90.0	86.0	62.0	60.0	185	112	124	79.0	199	177
Nb	1.1	0.90	4.2	4.1	3.1	2.9	3.2	2.0	1.1	1.4	5.8	3.5	3.1	1.5	4.8	8.2
Cs	0.38	0.53	1.5	0.31	0.79	0.52	0.59	0.96	0.37	0.56	1.5	0.91	0.91	0.46	1.6	0.30
Ba	199	205	676	164	419	310	323	234	168	241	461	292	296	229	467	583
La	4.20	3.61	10.2	6.49	6.52	5.85	7.02	3.83	3.99	5.52	16.0	10.2	10.5	6.47	17.0	19.1
Ce	12.7	10.9	22.4	19.1	16.6	15.2	18.1	9.79	11.4	13.5	41.0	25.9	27.0	16.3	41.9	46.3
Pr	1.78	1.54	3.32	2.73	2.83	2.34	2.59	1.69	1.79	1.92	5.77	3.77	4.37	2.72	6.42	6.43
Nd	8.49	7.80	13.5	13.1	12.7	12.0	13.1	10.9	8.85	9.75	26.5	18.1	20.1	13.6	29.0	28.2
Sm	2.56	2.44	2.89	3.86	3.25	3.36	3.91	2.53	2.90	3.10	6.42	4.61	5.29	3.85	7.14	6.27
Eu	0.850	0.830	0.953	1.14	1.08	1.14	1.27	0.719	1.01	0.991	1.82	1.41	1.46	1.12	1.84	1.79
Gd	2.69	2.67	2.58	4.04	3.12	3.28	3.53	3.01	2.55	2.90	5.17	3.78	4.58	3.56	6.22	5.36
Tb	0.470	0.470	0.359	0.695	0.465	0.540	0.630	0.424	0.497	0.589	0.955	0.723	0.750	0.580	0.970	0.791
Dy	3.06	3.05	2.28	4.37	3.08	3.33	3.88	2.79	3.20	3.35	5.88	4.48	4.25	3.50	5.66	4.69
Ho	0.690	0.670	0.550	0.941	0.763	0.740	0.750	0.625	0.626	0.623	1.17	0.932	0.840	0.730	1.17	0.924
Er	2.08	2.04	1.46	2.89	1.97	2.18	2.23	2.00	1.86	1.93	3.38	2.65	2.52	2.00	3.47	2.62
Tm	0.310	0.300	0.197	0.419	0.268	0.300	0.340	0.309	0.299	0.362	0.521	0.402	0.370	0.290	0.490	0.372
Yb	2.08	1.95	1.39	2.77	1.72	2.00	2.38	1.80	2.18	2.35	3.24	2.52	2.27	1.89	3.19	2.48
Lu	0.32	0.30	0.24	0.42	0.30	0.29	0.32	0.30	0.31	0.28	0.49	0.37	0.34	0.28	0.48	0.37
Hf	1.99	1.53	1.91	2.80	2.10	1.99	2.17	2.11	1.52	1.73	6.36	4.02	3.02	2.05	4.75	4.26
Ta	0.070	0.080	0.21	0.21	0.14	0.17	0.19	0.074	0.093	0.087	0.81	0.64	0.14	0.080	0.28	0.46
Tl	**0.048**	**0.046**	**0.27**	**0.049**	**0.088**	**0.12**	**0.095**	**0.079**	**0.029**	**0.033**	**0.12**	**0.049**	**0.046**	**0.058**	**0.061**	**0.11**
Pb	1.78	1.83	5.30	2.03	2.73	1.95	2.63	2.08	2.36	2.33	5.81	3.29	3.23	2.03	7.49	5.57
Th	0.60	0.48	3.2	0.59	1.6	1.0	0.91	0.84	0.28	0.61	2.2	1.4	0.95	0.49	2.3	1.5
U	0.28	0.23	1.5	0.38	0.77	0.49	0.61	0.42	0.18	0.44	1.2	0.72	0.65	0.32	1.3	0.59
$^{87}Sr/^{86}Sr$					0.703347	0.703352	0.703370	0.703482		0.703383	0.703374	0.703356	0.703341	0.703368	0.703331	0.703379
$^{143}Nd/^{144}Nd$					0.513048	0.513045	0.513047	0.513036		0.513032	0.513141	0.513081	0.513095	0.513077	0.513106	0.513046
$^{206}Pb/^{204}Pb$							18.320	18.301				18.185	18.192			
$^{207}Pb/^{204}Pb$							15.502	15.495				15.472	15.482			
$^{208}Pb/^{204}Pb$							38.033	38.037				37.850	37.885			
$\varepsilon^{205}Tl$	**-2.1**	**-1.5**	**-2.1**	**-4.7**	**-1.0**	**-4.1**	**-0.9**	**-2.5**	**+3.6**	**+0.9**	**+1.1**	**-0.1**	**+0.5**	**-0.8**	**-1.1**	**-1.7**

						**Esso**		**Achtang**			**Kluchevskoy**		**Bakening**		**Nikolka**	
	6250	ICH-96-19	ICH-96-28	ICH-96-31	ICH-96-54	ESO-96-08	ESO-96-04	ACH-96-01	ACH-96-02	ACH-96-03	KLU-96-03	KLU-96-12	BAK-95-17	BAK-95-34	8868	8878
	SR	SR	SR	SR	SR	SR	SR	SR	SR	SR	N-CKD	N-CKD	S-EVF	S-EVF	CKD	CKD
SiO_2	52.0	50.4	55.8	54.5	55.6	50.6	53.0	54.4	51.2	53.4	53.7	54.0	51.2	51.2	55.2	56.0
TiO_2	0.85	0.89	0.90	0.91	0.96	0.98	0.87	0.90	0.94	0.98	0.85	1.1	1.2	1.0	1.3	0.85
Al_2O_3	14.7	20.2	16.9	17.6	18.1	17.8	18.0	17.3	16.0	17.0	15.3	18.1	16.3	16.9	17.8	16.7
Fe_2O_3	3.01	3.11	1.89	2.49	2.22	2.39	2.27	2.28	2.94	2.31	2.06	2.89	2.01	2.08	4.42	1.78
FeO	5.62	5.08	5.18	4.91	4.77	6.43	5.73	5.19	5.84	5.77	6.42	5.61	6.52	6.70	4.12	5.70
MnO	0.16	0.14	0.13	0.13	0.13	0.16	0.15	0.15	0.16	0.15	0.16	0.16	0.16	0.17	0.14	0.14
MgO	8.41	4.88	4.97	4.60	4.22	6.51	5.48	5.13	7.89	6.15	8.88	5.03	8.29	7.80	2.46	4.19
CaO	10.1	9.77	7.71	8.08	7.92	9.75	8.55	7.62	9.61	8.19	9.40	8.14	8.90	8.94	7.18	7.40
Na_2O	2.66	3.17	3.57	3.47	3.73	2.92	3.17	3.28	2.86	3.19	2.89	3.60	3.38	3.18	3.48	3.30
K_2O	1.5	1.1	1.3	1.3	1.7	0.88	1.2	1.5	1.2	1.3	0.76	1.2	1.0	0.91	2.2	1.2
P_2O_5	0.23	0.25	0.32	0.30	0.38	0.23	0.22	0.31	0.29	0.28	0.16	0.22	0.29	0.23	0.33	0.17
LOI	0.63	0.40	0.51	0.69	1.0	0.59	0.67	1.1	0.66	0.76	0.33	0.40	0.60	0.47	2.2	1.1
Sc	32	25	22	24	22	30	27	23	30	22	36	21	29	29	22	29
V	243	219	172	199	179	261	231	196	243	216	240	259	236	237	282	227
Cr	346	33.0	97.0	66.0	48.0	186	121	99.0	337	163	426	31.0	349	272	202	330
Co	39	30	31	26	26	34	34	26	37	31	37	33	38	38	21	24
Ni	92.0	45.0	77.0	62.0	41.0	69.0	60.0	49.0	112	90.0	151	31.0	130	117	10.0	1.00
Rb	25	18	20	22	32	14	20	25	21	21	12	19	21	20	46	19
Sr	503	722	532	559	645	592	641	598	530	583	324	397	529	471	419	416
Y	18	15	18	16	18	19	18	22	21	21	15	22	23	24	27	18
Zr	95.0	93.0	157	128	163	70.0	79.0	122	93.0	115	81.0	104	108	92.0	142	93.0
Nb	3.7	3.6	6.0	5.6	7.0	1.7	3.1	6.4	3.9	5.5	1.2	2.0	4.7	3.6	3.0	1.7
Cs	0.38	0.21	0.40	0.49	0.75	0.36	0.46	0.44	0.41	0.36	0.42	0.51	0.64	0.58	2.0	0.24
Ba	443	454	469	449	575	369	493	576	373	461	290	434	346	304	743	478
La	9.03	8.22	16.5	13.2	16.9	6.11	7.89	12.8	8.76	10.6	5.41	8.43	11.0	9.40	14.4	8.12
Ce	22.2	19.0	36.4	28.7	37.0	17.7	21.3	33.1	24.0	28.5	13.6	21.1	26.7	22.7	35.7	19.4
Pr	3.30	2.86	4.69	4.03	4.93	2.54	2.80	4.21	3.27	3.72	2.17	3.39	4.04	3.40	5.28	2.98
Nd	15.2	14.0	20.7	19.1	23.2	12.1	12.4	17.8	14.7	16.1	10.4	15.8	18.6	15.7	24.7	14.2
Sm	3.82	3.55	5.13	4.55	5.15	3.15	3.04	4.02	3.62	3.77	2.94	4.14	4.65	4.00	6.33	3.67
Eu	1.11	1.19	1.43	1.29	1.42	1.02	0.950	1.20	1.11	1.14	0.904	1.27	1.39	1.24	1.65	1.12
Gd	3.31	3.12	4.38	4.12	4.41	2.85	2.60	3.47	3.30	3.34	2.89	3.77	4.05	3.70	5.41	3.26
Tb	0.520	0.490	0.730	0.620	0.690	0.445	0.390	0.510	0.500	0.470	0.451	0.631	0.653	0.610	0.880	0.540
Dy	3.10	2.79	4.15	3.42	3.69	2.64	2.32	3.02	3.04	2.87	2.87	3.84	3.82	3.60	3.14	5.33
Ho	0.610	0.550	0.780	0.670	0.720	0.535	0.480	0.629	0.640	0.610	0.607	0.816	0.749	0.720	0.980	0.640
Er	1.73	1.61	2.23	2.01	2.12	1.58	1.44	1.82	1.89	1.79	1.68	2.36	2.17	2.08	2.85	1.83
Tm	0.260	0.240	0.360	0.300	0.320	0.229	0.210	0.266	0.270	0.240	0.259	0.336	0.316	0.310	0.410	0.280
Yb	1.67	1.48	2.31	1.82	2.02	1.47	1.34	1.70	1.71	1.52	1.61	2.15	2.04	1.96	2.64	1.71
Lu	0.25	0.23	0.31	0.27	0.29	0.22	0.20	0.27	0.27	0.26	0.25	0.34	0.31	0.30	0.39	0.25
Hf	2.50	2.28	3.86	3.43	4.00	1.82	2.04	2.99	2.37	2.74	1.88	2.55	2.73	2.35	4.02	2.51
Ta	0.42	0.24	0.39	0.27	0.36	0.10	0.15	0.30	0.18	0.23	0.089	0.14	0.29	0.20	0.49	0.34
Tl	**0.12**	**0.033**	**0.10**	**0.10**	**0.18**	**0.084**	**0.11**	**0.14**	**0.10**	**0.11**	**0.051**	**0.078**	**0.075**	**0.083**	**0.081**	**0.20**
Pb	4.25	3.43	5.91	4.79	6.40	2.66	4.30	5.11	3.25	4.03	3.06	3.39	2.81	2.90	6.50	3.72
Th	1.5	0.76	1.8	1.8	2.6	0.38	0.69	1.2	0.71	1.0	0.61	0.80	1.4	1.5	1.7	0.65
U	0.71	0.39	0.84	0.77	1.2	0.26	0.40	0.63	0.38	0.47	0.41	0.49	0.60	0.56	1.2	0.51
$^{87}Sr/^{86}Sr$	0.703344	0.703329		0.703364		0.703355	0.703342	0.703354	0.703352	0.703295	0.703585	0.703664	0.703150		**0.703703**	**0.703597**
$^{143}Nd/^{144}Nd$	0.513077	0.513054		0.513041		0.513092	0.513063	0.513039	0.513044	0.513032	0.513080	0.513102	0.513070		**0.513113**	**0.513085**
$^{206}Pb/^{204}Pb$		18.248		18.248		18.249		18.199			18.281	18.303	18.298		**18.266**	**18.294**
$^{207}Pb/^{204}Pb$		15.497		15.488		15.486		15.460			15.500	15.509	15.493		**15.480**	**15.488**
$^{208}Pb/^{204}Pb$		37.953		37.975		37.916		37.841			37.971	37.997	37.998		**37.898**	**37.949**
$\varepsilon^{205}Tl$	**-2.0**	**-0.2**	**-1.5**	**-1.0**	**-1.3**	**-1.7**	**-1.5**	**-1.8**	**-1.7**	**-1.8**	**-0.6**	**-0.5**	**-0.7**	**-0.4**	**-1.1**	**-0.9**

	8883 CKD
SiO_2	51.1
TiO_2	1.4
Al_2O_3	18.4
Fe_2O_3	4.79
FeO	4.96
MnO	0.17
MgO	3.56
CaO	8.20
Na_2O	3.56
K_2O	1.4
P_2O_5	0.61
LOI	0.82
Sc	25
V	265
Cr	148
Co	28
Ni	22.0
Rb	41
Sr	469
Y	30
Zr	176
Nb	4.4
Cs	0.23
Ba	689
La	20.6
Ce	51.3
Pr	7.66
Nd	33.9
Sm	7.65
Eu	1.93
Gd	6.16
Tb	0.940
Dy	2.87
Ho	1.07
Er	3.05
Tm	0.440
Yb	2.86
Lu	0.43
Hf	4.38
Ta	0.50
Tl	**0.049**
Pb	7.19
Th	2.5
U	1.4
$^{87}Sr/^{86}Sr$	0.703441
$^{143}Nd/^{144}Nd$	0.513097
$^{206}Pb/^{204}Pb$	
$^{207}Pb/^{204}Pb$	
$^{208}Pb/^{204}Pb$	
$\varepsilon^{205}Tl$	**+0.5**

[a]Bold data of Sr, Nd and Pb isotopes and all thallium concentration and isotope compositions are from this study. Other data are from refs.[6,15]. Major elements (including LOI) in wt. % and trace elements in μg/g.

Supplementary Figures

Supplementary Fig. 1

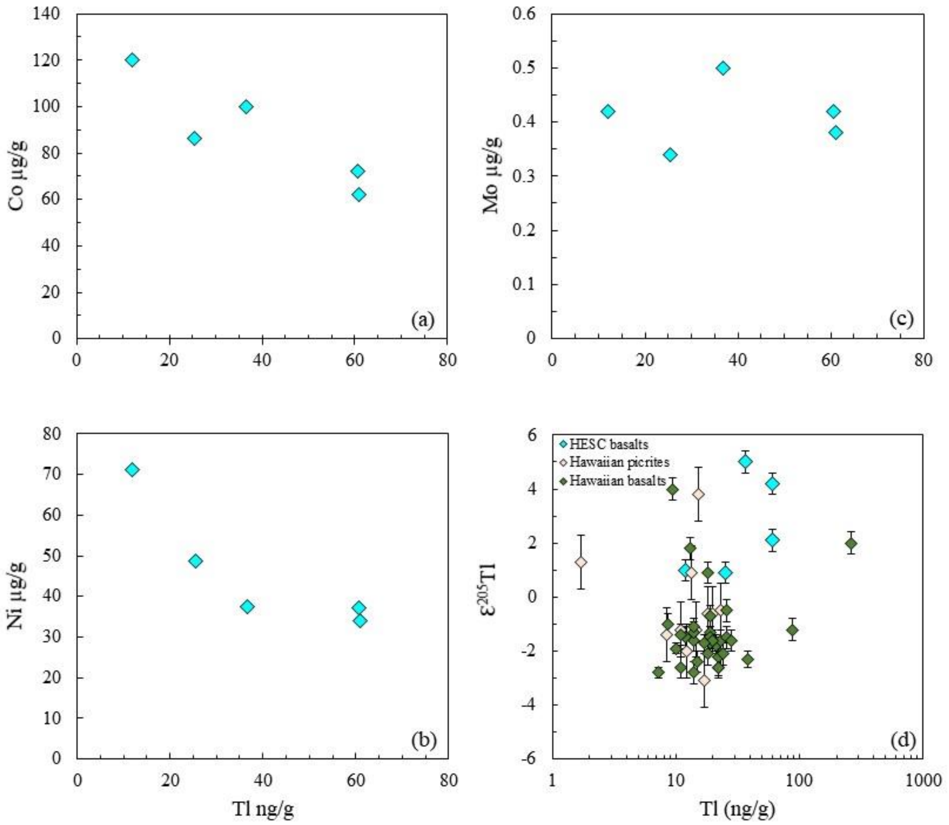

Supplementary Fig. 1 Thallium plotted against (a) Co, (b) Ni, (c) Mo concentrations, and (d) ε^{205}Tl values in Hawaii-Emperor Seamount Chain basalts. Tl concentrations in the samples show either negative relationship with Co and Ni concentrations or no relationship with Mo concentrations, which indicates that Tl contents in these samples are not related to direct precipitation of Mn oxides on the basalts, because Mn oxides usually contain ~5000 µg/g, ~2000 µg/g and ~500 µg/g of Co, Ni and Mo respectively[16,17]. Data of Hawaiian picrites and basalts are from the literature[13,18].

Supplementary Fig. 2

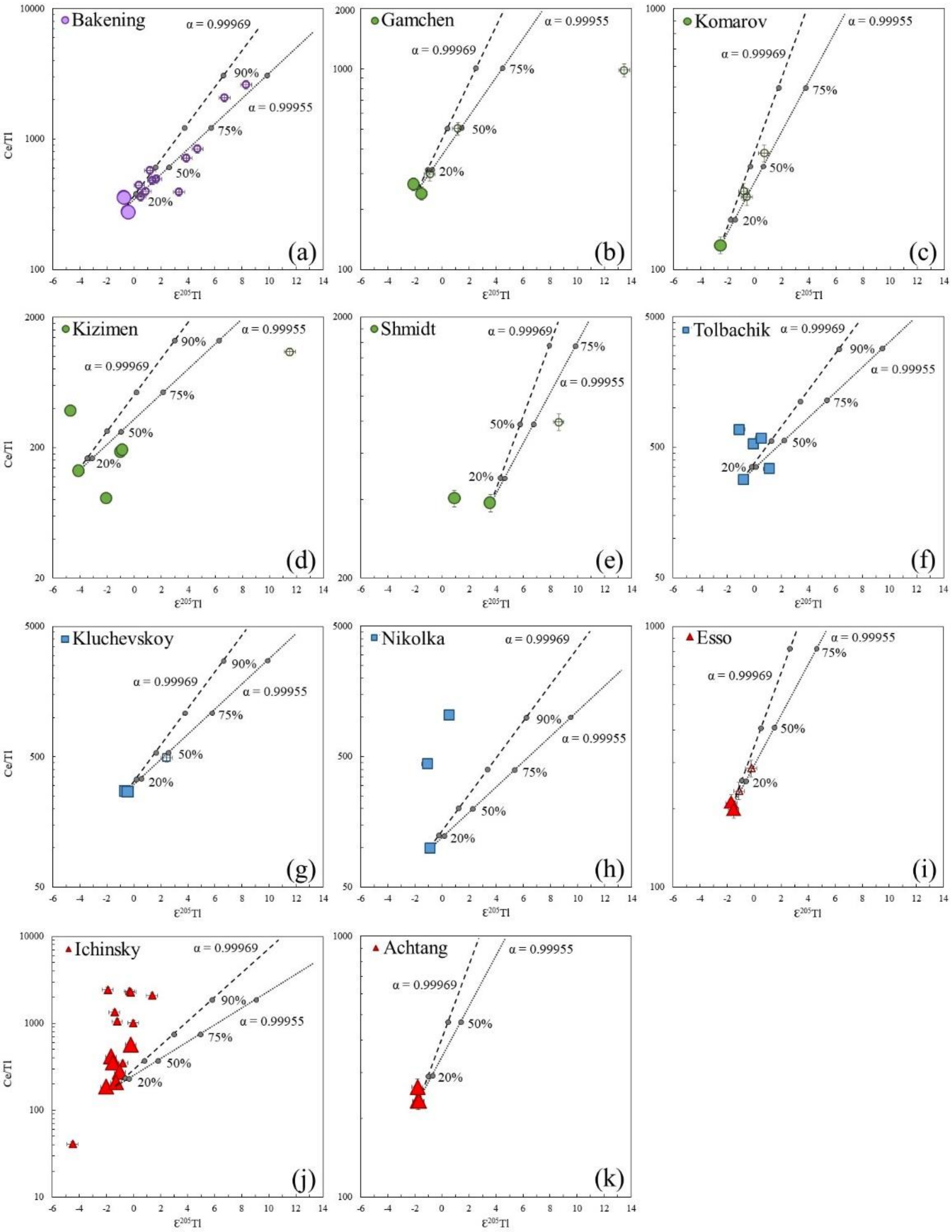

Supplementary Fig. 2 Tl isotope compositions plotted versus Ce/Tl for lavas from (a) Bakening, (b) Gamchen, (c) Komarov, (d) Kluchevskoy, (e) Esso, (f) Kizimen, (g) Shmidt, (h) Nikolka, (i) Tolbachik, (j) Ichinsky and (k) Achtang volcanos of Kamchatka arc. Variation of Tl isotope compositions and Ce/Tl ratios of lavas affected by magma degassing is simulated using two different kinetic models for natural (α = 0.99969) and forced (α = 0.99955) gas convection[3]. The starting composition of each volcano was estimated based on the least degassed samples. The numbers show Tl loss in percent relative to the modeled starting composition. Large symbols represent samples which were least affected by degassing.

Supplementary Fig. 3

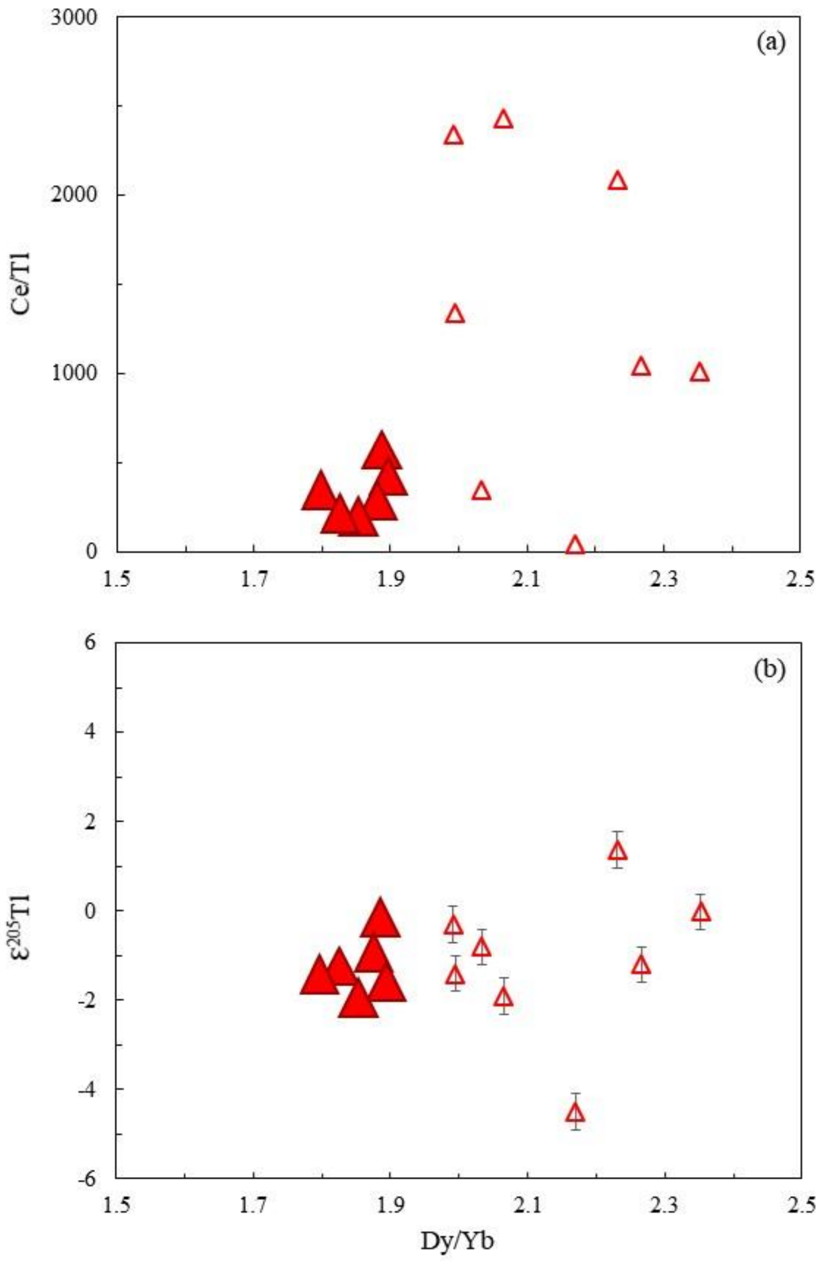

Supplementary Fig. 3 Dy/Yb plotted against (a) Ce/Tl and (b) Tl isotope compositions for Ichinsky volcano lavas (small open symbols). Large symbols represent Inchinsky lavas that show Dy/Yb and Ce/Tl similar to other lavas from other volcanoes of SR region and do not contain deep OIB components (or negligible amount).

Supplementary Fig. 4

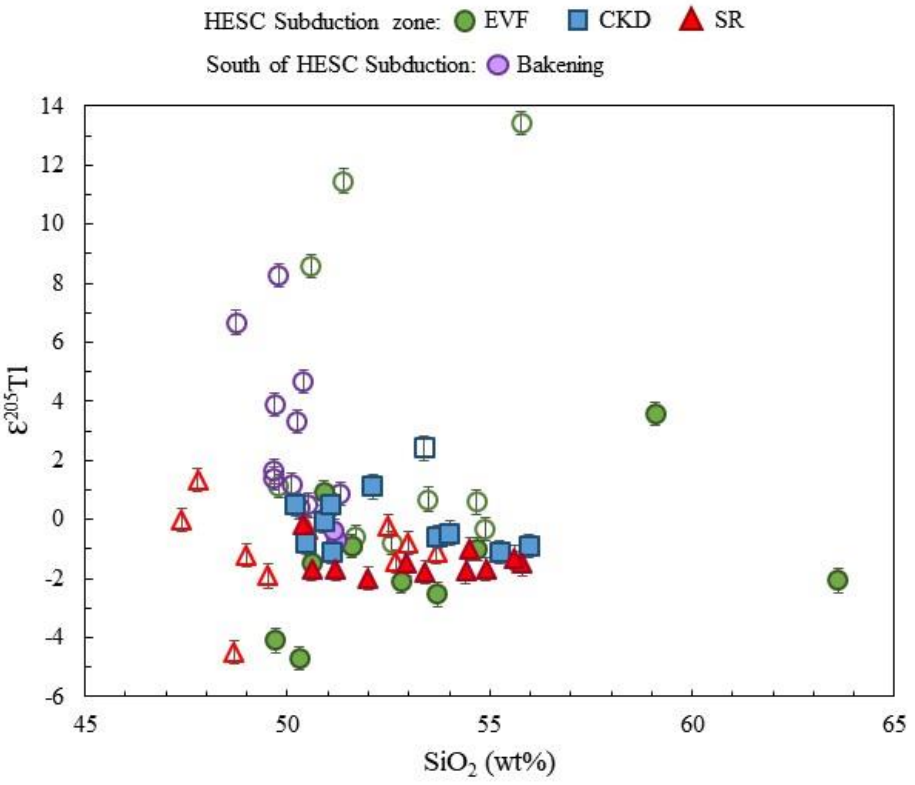

Supplementary Fig. 4 SiO_2 (wt%) plotted against ε^{205}Tl for all Kamchatka lavas. Open symbols represent filtered Kamchatka lavas for degassing and deep OIB components.

Supplementary Fig. 5

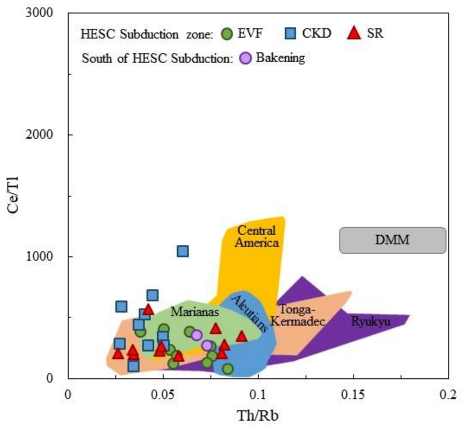

Supplementary Fig. 5 Plot of Th/Rb versus Ce/Tl ratios for the Kamchatka subaerial lavas. Also shown is the field of the DMM[19,20]. Unaltered arc lavas from Marianas[2], Aleutians[4], Tonga-Kermadec[21], Central America[21] and Ryukyu[5] are plotted for comparison.

Supplementary Fig. 6

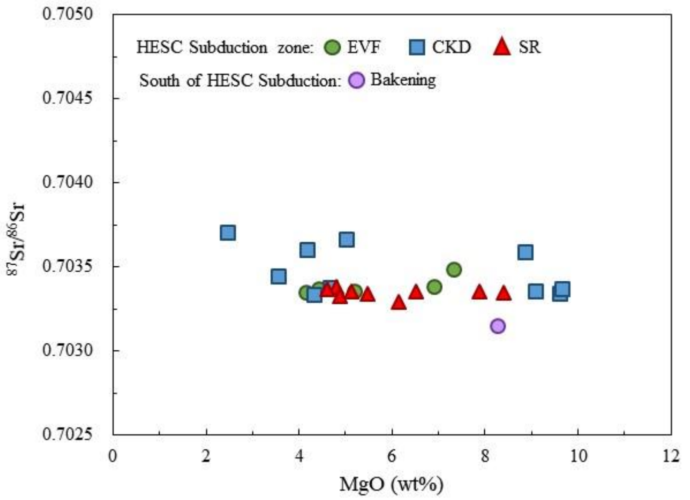

Supplementary Fig. 6 Plot of MgO (wt%) against $^{87}Sr/^{86}Sr$ for filtered Kamchatka lavas for degassing and deep OIB components. Data are from Supplementary Table 7.

Supplementary References

1 Baker, R. G. A., Rehkämper, M., Hinkley, T. K., Nielsen, S. G. & Toutain, J. P. Investigation of thallium fluxes from subaerial volcanism—Implications for the present and past mass balance of thallium in the oceans. *Geochimica et Cosmochimica Acta* **73**, 6340-6359, doi:10.1016/j.gca.2009.07.014 (2009).

2 Prytulak, J., Nielsen, S. G., Plank, T., Barker, M. & Elliott, T. Assessing the utility of thallium and thallium isotopes for tracing subduction zone inputs to the Mariana arc. *Chemical Geology* **345**, 139-149, doi:10.1016/j.chemgeo.2013.03.003 (2013).

3 Nielsen, S. G. *et al.* Thallium isotope fractionation during magma degassing: Evidence from experiments and Kamchatka arc lavas. *Geochemistry, Geophysics, Geosystems*, doi:10.1029/2020gc009608 (2021).

4 Nielsen, S. G. *et al.* Tracking along-arc sediment inputs to the Aleutian arc using thallium isotopes. *Geochimica et Cosmochimica Acta* **181**, 217-237, doi:10.1016/j.gca.2016.03.010 (2016).

5 Shu, Y. *et al.* Tracing subducted sediment inputs to the Ryukyu arc-Okinawa Trough system: Evidence from thallium isotopes. *Geochimica et Cosmochimica Acta* **217**, 462-491, doi:10.1016/j.gca.2017.08.035 (2017).

6 Churikova, T., Dorendorf, F. & Wörner, G. Sources and fluids in the mantle wedge below Kamchatka, evidence from across-arc geochemical variation. *Journal of Petrology* **42**, 1567-1593 (2001).

7 Sparks, R. Dynamics of magma degassing. *Geological Society, London, Special Publications* **213**, 5-22 (2003).

8 Feigenson, M. D., Hofmann, A. W. & Spera, F. J. Case studies on the origin of basalt. *Contributions to mineralogy and petrology* **84**, 390-405 (1983).

9 Schiano, P., Dupré, B. & Lewin, E. Application of element concentration variability to the study of basalt alteration (Fangataufa atoll, French Polynesia). *Chemical geology* **104**, 99-124 (1993).

10 Shaw, D. M. The geochemistry of thallium. *Geochimica et Cosmochimica Acta* **2**, 118-154 (1952).

11 Shannon, R. D. Revised effective ionic radii and systematic studies of interatomic distances in halides and chalcogenides. *Acta crystallographica section A: crystal physics, diffraction, theoretical and general crystallography* **32**, 751-767 (1976).

12 Heinrichs, H., Schulz-Dobrick, B. & Wedepohl, K. Terrestrial geochemistry of Cd, Bi, Tl, Pb, Zn and Rb. *Geochimica et Cosmochimica Acta* **44**, 1519-1533 (1980).

13 Nielsen, S. G., Rehkamper, M., Norman, M. D., Halliday, A. N. & Harrison, D. Thallium isotopic evidence for ferromanganese sediments in the mantle source of Hawaiian basalts. *Nature* **439**, 314-317, doi:10.1038/nature04450 (2006).

14 Prytulak, J. *et al.* Thallium elemental behavior and stable isotope fractionation during magmatic processes. *Chemical Geology* **448**, 71-83, doi:10.1016/j.chemgeo.2016.11.007 (2017).

15 Dorendorf, F., Wiechert, U. & Wörner, G. Hydrated sub-arc mantle: a source for the Kluchevskoy volcano, Kamchatka/Russia. *Earth and Planetary Science Letters* **175**, 69-86, doi:10.1016/s0012-821x(99)00288-5 (2000).

16 Gueguen, B. *et al.* Comparative geochemistry of four ferromanganese crusts from the Pacific Ocean and significance for the use of Ni isotopes as paleoceanographic tracers. *Geochimica et Cosmochimica Acta* **189**, 214-235, doi:10.1016/j.gca.2016.06.005 (2016).

17 Dutta, R., Sudarshan, M., Bhattacharyya, S., Chakravortty, V. & Chintalapudi, S. Quantitative PIXE analyses of ferromanganese oxide deposits from different locations of the Indian Ocean and a deposit from the Pacific Ocean. *Nuclear Instruments and Methods in Physics Research Section B: Beam Interactions with Materials and Atoms* **143**, 403-413 (1998).

18 Williamson, N. M. B., Weis, D. & Prytulak, J. Thallium isotopic compositions in Hawaiian lavas: evidence for recycled materials on the Kea side of the Hawaiian mantle plume. *Geochemistry, Geophysics, Geosystems*, doi:10.1029/2021gc009765 (2021).

19 Workman, R. K. & Hart, S. R. Major and trace element composition of the depleted MORB mantle (DMM). *Earth and Planetary Science Letters* **231**, 53-72, doi:10.1016/j.epsl.2004.12.005 (2005).

20 Nielsen, S. G., Shimizu, N., Lee, C. T. A. & Behn, M. D. Chalcophile behavior of thallium during MORB melting and implications for the sulfur content of the mantle. *Geochemistry, Geophysics, Geosystems* **15**, 4905-4919 (2014).

21 Nielsen, S. G. *et al.* Thallium isotopes as tracers of recycled materials in subduction zones: Review and new data for lavas from Tonga-Kermadec and Central America. *Journal of Volcanology and Geothermal Research* **339**, 23-40, doi:10.1016/j.jvolgeores.2017.04.024 (2017).
